# The association between nutrients and occurrence of COVID‐19 outcomes in the population of Western Iran: A cohort study

**DOI:** 10.1111/crj.13632

**Published:** 2023-05-11

**Authors:** Dana Vaisi, Farhad Moradpour, Shadieh Mohammadi, Daem Roshani, Yousef Moradi

**Affiliations:** ^1^ Department of Epidemiology and Biostatistics, Faculty of Medicine, School of Medicine Kurdistan University of Medical Sciences Sanandaj Iran; ^2^ Social Determinants of Health Research Center, Research Institute for Health Development Kurdistan University of Medical Sciences Sanandaj Iran; ^3^ Environmental Health Research Center, Research Institute for Health Development Kurdistan University of Medical Sciences Sanandaj Iran

**Keywords:** cohort, COVID‐19, macronutrients, micronutrients

## Abstract

**Introduction:**

The study aimed to determine the association between nutrients (micronutrients, macronutrients, and antioxidants) and the occurrence of COVID‐19‐related outcomes (morbidity and hospitalization) using a cohort study in Western Iran.

**Methods:**

The basic study information was collected from February 2019 to February 2020 from the baseline phase of the Dehgolan Prospective Cohort Study (DehPCS). The primary outcomes in this study included risk of contracting COVID‐19 and hospitalization due to it at a specific time. To compare these outcomes based on different nutritional groups (macronutrients or micronutrients), Kaplan–Meier chart and log rank test were used. Also, univariate and multivariate regression models were used to investigate the association between different nutritional groups and desired outcomes (risk of contracting COVID‐19 and hospitalization due to it at a certain time).

**Results:**

The results showed that people having an insufficient intake of selenium (HR: 1.180; % 95 CI: 1.032–2.490; P: 0.042), vitamin A (HR: 1.119; % 95 CI: 1.020–1.442; *p*: 0.033), and vitamin E (HR: 1.544; % 95 CI: 1.136–3.093; *p*: 0.039) were significantly more infected with COVID‐19 than the ones who had a sufficient intake of these nutrients. Also, the results showed that people having an insufficient intake of selenium (HR: 2.130; % 95 CI: 1.232–3.098; *p*: 0.018) and vitamin A (HR: 1.200; % 95 CI: 1.000–2.090; *p*: 0.043) were significantly hospitalized more than the ones who had a sufficient intake of these nutrients.

**Conclusion:**

Insufficient intake of selenium and vitamins A and E can significantly increase the incidence of COVID‐19 and hospitalization due to it.

## Graphical abstract

Low or abnormal intake of selenium and vitamins A and E can significantly increase the incidence of COVID‐19 and hospitalization due to it.

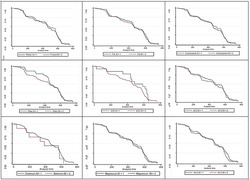

## 1 INTRODUCTION

COVID‐19 is an emerging infectious disease caused by SARS‐CoV‐2 with a pandemic capability in humans, which has become a major global threat. Currently, the existence of different pathogenic subgroups of this virus and renewed pandemics in the future are among the main concerns related to this disease. Having a properly functioning immune system is necessary to defend the host against these pathogenic microorganisms. Therefore, effective methods to prevent and control this infectious disease are very important.^1^ Both the immune system and the digestive system have important tasks against the infection of COVID‐19 and to fight against this virus. Therefore, the body should be evaluated in terms of sufficient intake of nutrients (macronutrients and micronutrients).^2^ The nutritional status of populations can be recognized as a key factor affecting resilience against the instability caused by the current COVID‐19 pandemic.^3^ One of the most basic questions is “What is the nutrition role in this regard?” and “What role do food and food supplements play in preventing the disease or recovering from it?” Proper nutrition is the first line of treatment for people suffering from COVID‐19. In addition to being of great importance in dealing with this infection, it is also effective in preventing the spread of other diseases. Nutrition always affects the proper functioning of the immune system and determines the risk and severity of infections. There are two‐way associations between diet, infection, and immunity.^4^ Macronutrients and micronutrients, especially in colorful fruit and vegetables, promote healthy immune system responses. These micronutrients, antioxidants, and anti‐inflammatory nutrients including beta‐carotene, vitamins C and E, and polyphenolic compounds regulate immune system function. Anti‐inflammatory strategy, whether through food, nutrients, or drugs, is considered a suitable option for managing COVID‐19.^5^ The impact of nutrients such as macronutrients or micronutrients in managing and controlling the COVID‐19 disease is undeniable. Selenium is one of these essential minerals for the body, playing a prominent role in defense against viral infections.^6^, ^7^ Selenium deficiency may cause lung damage.^7^ On the other hand, selenium in combination with vitamin E prevents the formation of free radicals and oxidative damage to cells and tissues.^8^ Another mineral affecting the reduction in COVID‐19 infection is magnesium, which affects both cellular and humoral immunity,^9^ and its deficiency increases the risk of frequent upper respiratory tract infections. Vitamin D is involved in a wide range of immune system regulatory activities.^10^ Low levels of vitamin D increase the risks, severity, complications, and mortality of several respiratory diseases such as asthma and so forth. Vitamin C is known for its role in collagen synthesis of connective tissues and acts as an antioxidant. This vitamin also supports various functions of the body's immune system and protects the body against infection caused by COVID‐19.^11^ Also, studies showed that the lack of some substances such as vitamin E and selenium causes some genetic mutations, which significantly increases the virulence of viruses in people. As the nutrition role is evident in all diseases, including cancer, cardiovascular, and metabolic ones, there is no doubt that nutrition plays an essential role in infectious diseases, especially in COVID‐19.^12^ Therefore, the lack of nutrients such as zinc, selenium, iron, vitamins A, B, C, D, and E, proteins, as well as carbohydrates can affect the body's immune responses and the function of the immune system and also make people susceptible to infectious diseases such as COVID‐19.^13^ The study aimed to determine the association between nutrients (insufficient or sufficient intake of micronutrients, macronutrients and antioxidants) and the occurrence of COVID‐19‐related outcomes (morbidity and hospitalization) using the Dehgolan Prospective Cohort Study (DehPCS).

## 2 METHODS

### 2.1 Study design and participants

The present research was a prospective cohort study profiting from the data of DehPCS. DehPCS is one of the 18 prospective epidemiological cohort studies in Iran (PERSIAN), which is being performed on the population of 35–70 years old, permanent residents of Dehgolan with the aim of assessing the risk factors of common non‐communicable diseases in the region. All PERSIAN sites use the same protocol to conduct the study. The study design and sampling method were previously discussed in detail in the related published article.^14^ Briefly, participants from the general population, aged 35 to 70 years, were entered in the study through notifications and direct contacts of the researchers. The basic study information including age (four age groups: 35–44, 45–54, 55–64, and 65 years and over), marital status (single or married), education level (literate or illiterate), gender (female or male), body mass index (normal or abnormal), waist circumference (normal or abnormal), cardiovascular diseases, diabetes, stroke, heart attack, and systolic or diastolic blood pressure was collected from February 2019 to February 2020. The data related to COVID‐19 were collected from January 2021 to December 2022. The study inclusion criteria were registration in the registry or baseline data phase in DehPCS and the presence of complete desired information, especially individuals' nutritional characteristics in the cohort documents. Exclusion criteria included missing information in the baseline data for participants in the cohort study.

### 2.2 Dietary assessment

A semi‐quantitative food frequency questionnaire (FFQ) containing 120 questions in a valid block format was used to collect nutritional data in this study, and it was completed based on the participants' diet in the last 12 months because it has been designed for long‐term dietary assessment. This questionnaire is specially designed for Iranian adults.^15^ Trained interviewers evaluated people's diet based on their answers in three steps. In the first step, the interviewer recorded the number of times each food item was consumed by each participant. In the second step, the consumption amount was recorded for each time. In the third step, the number of consumption months during the last year was recorded for each food item. To determine the consumption of each food item, the questioner used common household scales to increase the measurement accuracy. Finally, based on the number of consumption times, the amount of consumption for each time and the number of months of consumption of each food item during the last year, the intake amount of each food item was calculated based on grams per day. The daily intake of nutrients was also calculated for each person. The Food and Nutrient Database of the United States Department of Agriculture (USDA) was used in adjusted dietary studies for Iranian foods to extract nutrient content.^16^ Previous studies showed FFQs could reasonably be used for long‐term diet assessment.^17^

### 2.3 Definition and classification of nutrients

In the definition and classification of nutrients for each of the nutrients including micronutrients (vitamins A, E, C, and D, magnesium, and selenium) and macronutrients (proteins, carbohydrates, and fats), the required cut‐off was determined in men and women and classified into two groups of sufficient and insufficient intake. For this purpose, the permissible and non‐permissible daily intake of each micronutrient and macronutrient in women and men was determined according to international nutrition standards and valid references. For vitamin A, based on the intake reference, the recommended limit for men and women is 3000 mcg (10 000 IU) per day.^18^, ^19^ An intake more and less than the determined values by people was considered as insufficient intake in the study. The recommended daily intake of vitamin E for men and women is 15 mg per day. An intake more and less than the determined values by people was considered as insufficient intake in the study.^18^, ^19^ The recommended daily intake of vitamin C for men and women is 90 and 75 mg per day, respectively. An intake more and less than the determined values by people was considered as insufficient intake in the study.^18^, ^19^ The recommended daily intake of vitamin D in men and women aged 31–70 years is 15 mcg per day, equivalent to 600 IU.^18^, ^19^ An intake more and less than the determined values by people was considered as insufficient intake in the study. The recommended daily intake of magnesium for men over 18 years old is 420 mg per day and for women over 18 years old is 320 mg per day. There is no limit to its intake from food for men and women, but the maximum daily intake of magnesium from medicine and supplements is recommended to be up to 350 mg per day.^18^, ^19^ An intake more and less than the determined values by people was considered as insufficient intake in the study. According to the reference, the permissible limit of selenium in men and women is up to 55 mcg per day.^18^, ^19^ An intake more and less than the determined values by people was considered as insufficient intake in the study. For macronutrients, it is suggested that 25%–35% of daily calories or energy intake should come from fats, 55%–65% from carbohydrates, and 10%–15% from proteins. As each gram of protein and carbohydrate produces 4 kcal of energy and each gram of fats produces 9 kcal of energy, to obtain the cut‐off of these macronutrients (proteins, carbohydrates, and fats), participants' daily protein and carbohydrate intake was multiplied by the number 4 and their daily fat intake by the number 9. Then, the result was divided by the people's daily energy intake, and finally, the result was multiplied by 100. In this way, the cut‐off determined for each of the macronutrients was obtained for the people participating in the study. If the numbers obtained from this multiplication and division for proteins were in the range of 10%–15%, the people had a sufficient intake, and outside the mentioned range (an intake less than 10% and more than 15%) the people had an insufficient intake. For carbohydrates, if obtained numbers were in the range of 55% to 65%, they were considered as sufficient intake by the people participating in the study, and outside the mentioned range (intake less than 55% and more than 65%), they were insufficient intake. For fats, if obtained numbers were in the range of 25% to 35%, people were classified in a sufficient intake category, and otherwise (less than 25% and more than 35%), they were classified in an insufficient intake category. Because fiber is considered a type of carbohydrate, its daily intake in people's diet was categorized into two groups of insufficient and sufficient intake. Based on the relevant references, the recommended amount of fiber intake in women aged less than and over 50 years is 21 to 25 and 25 to 30 g per day, respectively, and in men aged less than and over 50 years, the recommended amount of fiber intake is 30 to 38 and 38 to 45 g per day, respectively. Based on these determined ranges, people were divided into two groups of insufficient and sufficient intake. Regarding individuals' cholesterol intake, the intake up to 300 mg per day was considered as normal and more than the mentioned number was considered as insufficient. Based on the references related to the daily intake of total fatty acids, the maximum permissible amount of intake is between 55 to 77 g per day for adults. In the present study, an intake greater than 77 g and lower than 55 g were considered insufficient.

### 2.4 Outcomes

The primary outcome in this study included the symptom‐based report of the disease in the study participants and then the definitive diagnosis of COVID‐19 in people with symptoms through polymerase chain reaction (PCR) testing. For this purpose, first the participants were checked for sudden onset of fever above 38.5 degrees and cough. Then, by linking the information available in the disease control and prevention group of Kurdistan province and Iran and the database of the reference laboratory for the diagnosis of COVID‐19 in Kurdistan province with the cohort study data through individuals' national code, their definitive infection status was checked. Hospitalized cases were also examined using hospital data at the province level, and information was extracted along with the national code of all people hospitalized in the province because of being infected with COVID‐19. Then, this information was merged with the cohort data using the participants' national code, and the cohort inpatient cases were also determined.

### 2.5 Statistical analysis

In this study, raw frequency along with percentages of desired variables were used to perform descriptive analysis. The intended outcomes in this study were the risk of contracting COVID‐19 and hospitalization due to it at a specific time. To report and compare these outcomes in the studied cohort community based on different nutritional groups (macronutrients or micronutrients), Kaplan–Meier chart and log rank test were used. Also, univariate and multivariate regression models were used to investigate the association between different nutritional groups (macronutrients or micronutrients) and desired outcomes (risk of contracting COVID‐19 and hospitalization due to it at a certain time). In the multivariate model, the variables of age, education, gender, marital status, body mass index, and waist circumference were controlled or adjusted.

The stepwise regression analysis was applied in the multiple Cox regression and the multicollinearity problem was completely checked for final selection. The proportional hazard (PH) assumption was tested based on the scaled Schoenfeld residuals for all variables in the Cox regression. All analyses were performed using the STATA version 17. A *p*‐value of less than 0.05 was considered significant.

## 3 RESULTS

The demographic and clinical characteristics of the participants in the current cohort study are shown in Table 1. The results showed that most of the participants in the study were female (2228 people or 55.76%), literate (2219 people or 55.92%), married (3651 people or 92.1%), and in the age group of 35 to 44 years (1597 people or 40.25%). Also, from all the people participating in the study, 976 (24.70%) had a normal body mass index, 953 (24.11%) had a normal waist circumference, 2689 (67.99%) had a normal cholesterol intake, 2944 (74.44%) had a sufficient intake of saturated fatty acids, 2135 (54.19%) had normal systolic blood pressure, and 3341 (84.40%) had normal diastolic blood pressure. In terms of underlying diseases, 522 and 300 people had diabetes and cardiovascular diseases, respectively (Table 1). From the total population of DehPCS, 270 people (6.75%) had a positive PCR test whereas 259 (6.49%) were hospitalized due to Covid‐19 (Table 1).

**TABLE 1:** The baseline and characteristics of all study population in DehPCS.

Variables	No. (percent)	Variables	No. (percent)
Sex		Received cholesterol	
Male	1768 (44.24%)	Sufficient intake	2689 (67.99%)
Female	2228 (55.76%)	Insufficient intake	1266 (32.01%)
Education		Saturated fatty acid	
Illiterate	1749 (44.08%)	Sufficient intake	2944 (74.44%)
Literate	2219 (55.92%)	Insufficient intake	1011 (25.56%)
Marital status		SBP	
Single	40 (1.01%)	Normal	2135 (54.19%)
Married	3956 (98.99%)	Abnormal	1805 (45.81%)
Age groups		DBP	
35–44 Years	1597 (40.25%)	Normal	3341 (84.40%)
45–54 Years	1381 (34.80%)	Abnormal	599 (15.20%)
64–55 Years	755 (19.03%)		
≥65 Years	235 (5.92%)		
BMI		Diabetes	
Normal	976 (24.70%)	No	3426 (86.78%)
Abnormal	29.76 (75.30%)	Yes	522 (13.22%)
Waist circumference		Cardiovascular diseases	
Normal	953 (24.11%)	No	3648 (92.40%)
Abnormal	2976 (75.89%)	Yes	300 (7.60%)
Myocardial infraction		Stroke	
No	3875 (98.15%)	No	3887 (98.45%)
Yes	73 (1.73%)	Yes	61 (1.55%)
COVID‐19 test		COVID‐19 admission	
Positive	270 (6.75%)	No	3737 (93.51%)
Negative	3726 (93.25%)	Yes	259 (6.49%)

Abbreviations: BMI, body mass index; DBP, diastolic blood pressure; DehPCS, Dehgolan Prospective Cohort Study; SBP, systolic blood pressure.

In total, from the people participating in DehPCS, 3214 (81.37%), 1012 (25.62%), 1723 (43.62%), and 781 (19.75%) had a sufficient intake of proteins, fats, carbohydrates, and fiber, respectively. Regarding the intake of micronutrients, the results showed that most of the participants had a sufficient intake, except for vitamins D, E, and A, which the majority of the participants did not consume in sufficient amounts (Table 2).

**TABLE 2:** The frequency of macro and micronutrients status (sufficient and insufficient intake) in DehPCS.

Variables	No. (percent)	Variables	No. (percent)
Received protein		Received vitamin A (mcg)	
Sufficient intake	3214 (81.37%)	Sufficient intake	1140 (28.82%)
Insufficient intake	736 (18.63%)	Insufficient intake	2815 (71.18%)
Received fatty		Received vitamin A (IU)	
Sufficient intake	1012 (25.62%)	Sufficient intake	2734 (69.13%)
Insufficient intake	2938 (74.38%)	Insufficient intake	1221 (30.87%)
Received carbohydrate		Received vitamin E	
Sufficient intake	1723 (43.62%)	Sufficient intake	170 (4.30%)
Insufficient intake	2227 (56.38%)	Insufficient intake	3785 (95.70%)
Received fiber		Received vitamin D (IU)	
Sufficient intake	781 (19.75%)	Sufficient intake	1 (0.03%)
Insufficient intake	3174 (80.25%)	Insufficient intake	3954 (99.97%)
Received magnesium		Received vitamin D (mcg)	
Sufficient intake	2173 (54.94%)	Sufficient intake	1 (0.03%)
Insufficient intake	1782 (45.06%)	Insufficient intake	3954 (99.97%)
Received selenium		Received vitamin C	
Sufficient intake	3853 (97.42%)	Sufficient intake	2818 (71.25%)
Insufficient intake	102 (2.58%)	Insufficient intake	1137 (11.37%)

Abbreviation: DehPCS, Dehgolan Prospective Cohort Study.

**FIGURE 1:**
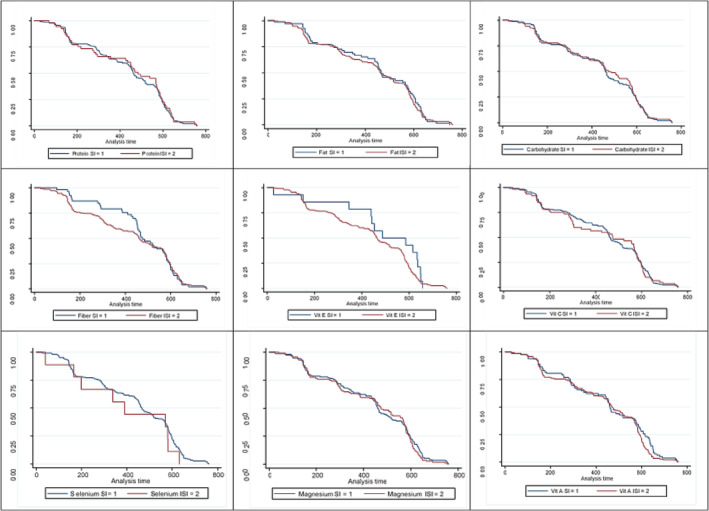
The survival rate in patients with coronavirus disease 2019 (COVID 19) positive test based on received protein (the log rank test: 1.33, df: 1, *p*: 0.281), fatty (the log rank test: 2.58, df: 1, *p*: 0.121), carbohydrates (the log rank test: 0.58, df: 1, *p*: 0.330), fiber (the log rank test: 1.33, df: 1, *p*: 0.445), vitamin E (the log rank test: 2.31, df: 1, *p*: 0.770), vitamin C (the log rank test: 2.22, df: 1, *p*: 0.763), selenium (the log rank test: 1.89, df: 1, *p*: 0.330), magnesium (the log rank test: 2.01, df: 1, *p*: 0.449), and vitamin A (the log rank test: 2.92, df: 1, *p*: 0.232). SI, sufficient intake; ISI, insufficient intake.

The first outcome in the present cohort study was the infection with COVID‐19, and the association of the desired variables with this outcome has been reported in Table 3. The results showed people having an insufficient intake of selenium and vitamins A and E were significantly more infected with COVID‐19 than the ones who had a sufficient intake of these nutrients. The results indicated that the instantaneous hazard ratio in the univariate model for contracting COVID‐19 in people having an insufficient selenium intake compared with the ones who had a sufficient intake of this nutrient in their diets was 1.343 times (HR: 1.343; % 95 CI: 1.110–2.420; P: 0.030). This association in the multivariate model with the control of confounding variables was equal to 1.180 (HR: 1.180; % 95 CI: 1.032–2.490; P: 0.042) (Table 3 and Figure 1).

**TABLE 3:** Multivariable and univariate cox regression analysis of demographic, clinical, macro, and micronutrients associated with COVID‐19 test positive in DehPCS.

Variables	Univariate model			Multivariate model		
	HR	% 95 CI	*p* value	HR	% 95 CI	*p* value
Sex						
Male	1			1		
Female	0.886	0.691–1.110	0.339	0.978^a^	0.770–1.244	0.862
Education						
Illiterate	1			1		
Literate	0.684	0.391–1.190	0.183	0.684^b^	0.391–1.190	0.183
Marital Status						
Single	1			1		
Married	2.810	0.385–20.590	0.307	2.009^c^	0.667–18.880	0.490
Age groups						
35–44 years	1			1		
45–54 years	0.963	0.716–1.290	0.804	1.177^d^	0.882–1.570	0.267
64–55 years	1.189	0.846–1.650	0.323	1.040^d^	0.757–1.440	0.772
≥65 years	1.570	0.979–2.520	0.061	1.740^d^	1.100–2.750	0.018
BMI						
Normal	1			1		
Abnormal	0.948	0.716–1.250	0.713	0.997^e^	0.742–1.332	0.984
Waist circumference						
Normal	1			1		
Abnormal	0.980	0.688–1.230	0.577	0.997^f^	0.658–1.220	0.492
SBP						
Normal	1			1		
Abnormal	1.050	0.824–1.340	0.684	1.060^g^	0.610–1.400	0.624
DBP						
Normal	1			1		
Abnormal	1.030	0.717–1.499	0.854	1.065^g^	0.729–1.540	0.751
Diabetes						
No	1			1		
Yes	0.968	0.689–1.360	0.856	0.934^g^	0.657–1.320	0.706
Cardiovascular diseases						
No	1			1		
Yes	0.807	0.551–1.188	0.273	0.717^g^	0.472–1.080	0.119
Stroke						
No	1			1		
Yes	1.680	0.745–3.780	0.210	1.200^g^	0.520–2.860	0.675
Myocardial infraction						
No	1			1		
Yes	0.884	0.329–2.370	0.808	0.788^g^	0.276–2.240	0.657
Received protein						
Sufficient intake	1			1		
Insufficient intake	0.966	0.714–1.300	0.823	0.862^g^	0.628–1.180	0.357
Received fatty						
Sufficient intake	1			1		
Insufficient intake	1.020	0.778–1.350	0.842	1.120^g^	0.839–1.510	0.423
Received carbohydrate						
Sufficient intake	1			1		
Insufficient intake	0.986	0.760–1.230	0.799	0.939^g^	0.723–1.220	0.642
Received fiber						
Sufficient intake	1			1		
Insufficient intake	1.050	0.780–1.420	0.726	1.030^g^	0.751–1.410	0.846
Received magnesium						
Sufficient intake	1			1		
Insufficient intake	1.199	1.000–1.410	0.049	1.080^g^	0.845–1.390	0.519
Received selenium						
Sufficient intake	**1**			**1**		
Insufficient intake	**1.343**	**1.110–2.420**	**0.030**	**1.180** ^**g**^	**1.032–2.490**	**0.042**
Received vitamin A (mcg)						
Sufficient intake	**1**			**1**		
Insufficient intake	**1.190**	**1.000–1.553**	**0.043**	**1.119** ^**g**^	**1.020–1.442**	**0.033**
Received vitamin E						
Sufficient intake	**1**			**1**		
Insufficient intake	**1.389**	**1.042–2.370**	**0.030**	**1.544** ^**g**^	**1.136–3.093**	**0.039**
Received vitamin D (mcg)						
Sufficient intake	1					
Insufficient intake	NR	‐	‐	‐	‐	‐
Received vitamin C						
Sufficient intake	1			1		
Insufficient intake	1.010	0.759–1.389	0.932	1.059^g^	0.777–1.440	0.713

Abbreviations: BMI, body mass index; DBP, diastolic blood pressure; DehPCS, Dehgolan Prospective Cohort Study; SBP, systolic blood pressure.

^a^ Adjusted based on education level, marital status, age, BMI, and waist circumference.

^b^ Adjusted based on sex, marital status, age, BMI, and waist circumference.

^c^ Adjusted based on education level, sex, age, BMI, and waist circumference.

^d^ Adjusted based on education level, marital status, sex, BMI, and waist circumference.

^e^ Adjusted based on education level, marital status, age, sex, and waist circumference.

^f^ Adjusted based on education level, marital status, age, BMI, and sex.

^g^ Adjusted based on education level, marital status, age, BMI, sex, and waist circumference.

The results showed the instantaneous hazard ratio in the univariate model for contracting COVID‐19 in people having an insufficient intake of vitamin A was 1.190 times compared with people who had a sufficient intake of this nutrient in their diet (HR: 1.190; 95% CI: 1.000–1.553; *p*: 0.043). This association in the multivariate model with the control of confounding variables was equal to 1.119 (HR: 1.119; % 95 CI: 1.020–1.442; *p*: 0.033) (Table 3 and Figure 1). Also, the instantaneous hazard ratio in the univariate model for contracting COVID‐19 in people having an insufficient intake of vitamin E was 1.389 times compared with people who had a sufficient intake of this nutrient in their diet (HR: 1.389; % 95 CI: 1.042–2.370; *p*: 0.030). This association in the multivariate model with the control of confounding variables was equal to 1.544 (HR: 1.544; % 95 CI: 1.136–3.093; *p*: 0.039) (Table 3 and Figure 1).

**FIGURE 2:**
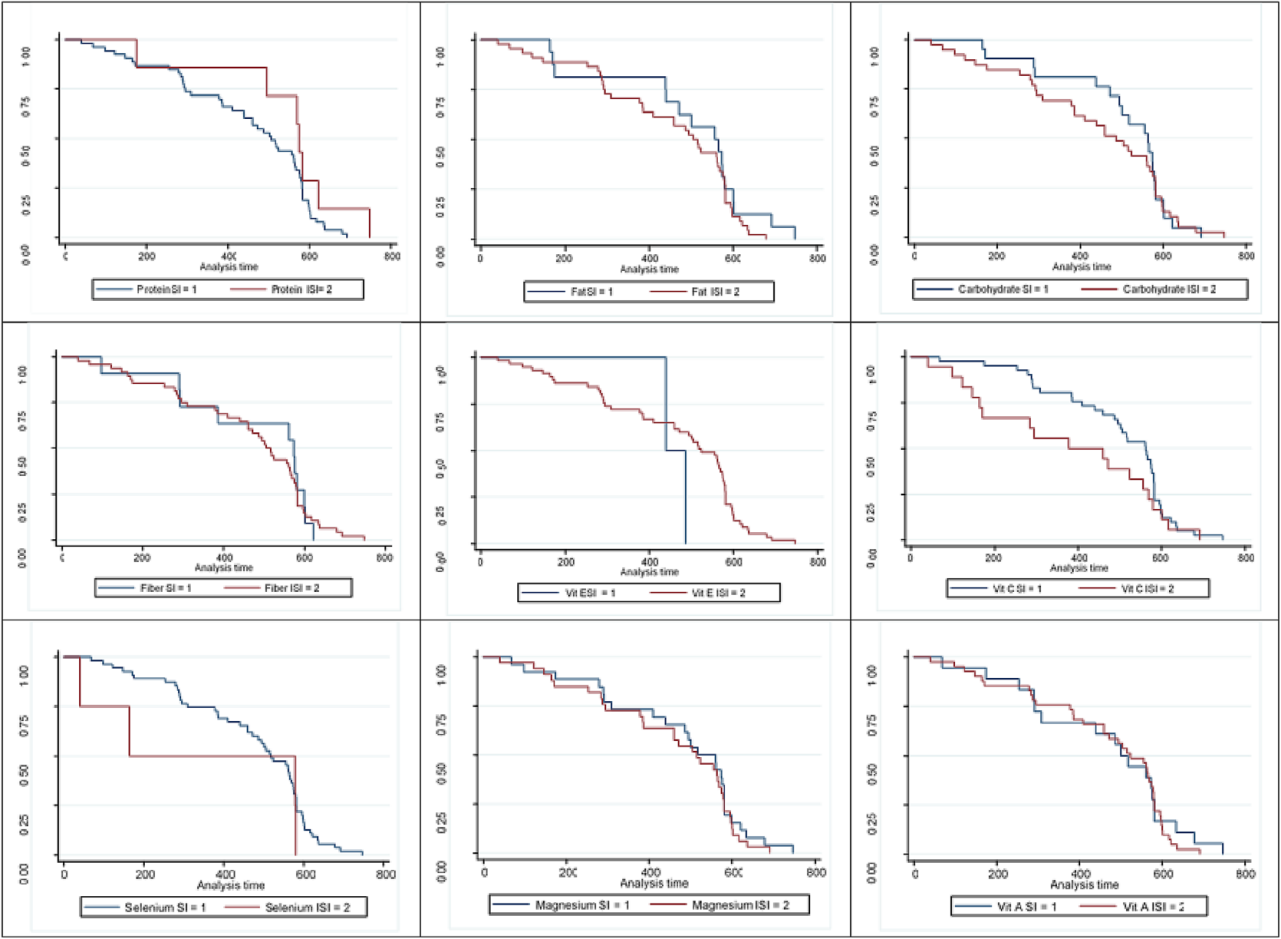
The survival rate in patients with coronavirus disease 2019 (COVID 19) admission based on received protein (the log rank test: 1.39, df: 1, *p*: 0.299), fatty (the log rank test: 2.29, df: 1, *p*: 0.201), carbohydrates (the log rank test: 0.88, df: 1, *p*: 0.509), fiber (the log rank test: 1.09, df: 1, *p*: 0.402), vitamin E (the log rank test: 1.98, df: 1, *p*: 0.708), vitamin C (the log rank test: 2.09, df: 1, *p*: 0.652), selenium (the log rank test: 1.97, df: 1, *p*: 0.331), magnesium (the log rank test: 2.21, df: 1, *p*: 0.390), and vitamin A (the log rank test: 2.98, df: 1, *p*: 0.239). SI: sufficient intake, ISI: insufficient intake.

The second outcome in the current cohort study was hospitalization due to COVID‐19, and the association of the desired variables with this outcome has been reported in Table 4. The results showed that people having an insufficient intake of protein, selenium, and vitamin A were significantly hospitalized more than ones who had a sufficient intake of these nutrients. The results indicated that the instantaneous hazard ratio in the univariate model for hospitalization due to COVID‐19 in people having an insufficient intake of selenium was 1.610 times compared with people who had a sufficient intake of this nutrient in their diet (HR: 1.610; % 95 CI: 1.054–2.740; *p*: 0.009). This association in the multivariate model with the control of confounding variables was equal to 2.130 (HR: 2.130; % 95 CI: 1.232–3.098; *p*: 0.018) (Table 4 and Figure 2). The results showed that the instantaneous hazard ratio in the univariate model for hospitalization due to COVID‐19 in people having an insufficient intake of vitamin A was 1.150 times compared with ones who had a sufficient intake of this nutrient in their diet (HR: 1.150; 95% CI: 1.001–1.908; *p*: 0.044). This association in the multivariate model with the control of confounding variables was equal to 1.200 (HR: 1.200; % 95 CI: 1.000–2.090; *p*: 0.043) (Table 4 and Figure 2). Also, the instantaneous hazard ratio in the univariate model for hospitalization of people having an insufficient intake of protein was 48% lower than that of participants who had a sufficient intake of this nutrient in their diet, but it was not statistically significant in this model (HR: 0.525; % 95 CI: 0.124–1.230; *p*: 0.140). This association in the multivariate model with the control of confounding variables was equal to 0.238, which was statistically significant (HR: 0.238; % 95 CI: 0.027–0.687; *p*: 0.008) (Table 4 and Figure 2).

**TABLE 4:** Multivariable and univariate Cox regression analysis of demographic, clinical, macro, and micronutrients associated with COVID‐19 admission in DehPCS.

Variables	Univariate model			Multivariate model		
	HR	% 95 CI	*p* value	HR	% 95 CI	*p* value
Sex						
Male	1			1		
Female	0.703	0.412–1.200	0.197	0.982^a^	0.399–1.866	0.686
Education						
Illiterate	1			1		
Literate	0.968	0.327–2.860	0.945	0.889^b^	0.302–2.611	0.832
Marital status						
Single	1			1		
Married	1.334	1.102–3.093	0.033	1.893^c^	1.001–3.439	0.020
Age groups						
35–44 years	1			1		
45–54 years	0.989	0.469–2.089	0.978	0.989^d^	0.469–2.089	0.978
64–55 years	0.789	0.396–1.577	0.502	0.789^d^	0.396–1.577	0.502
≥65 years	2.160	0.897–5.220	0.086	2.160^d^	0.897–5.220	0.086
BMI						
Normal	1			1		
Abnormal	0.571	0.299–1.090	0.090	0.808^e^	0.346–1.880	0.623
Waist						
Normal	1			1		
Abnormal	0.495	0.256–0.960	0.038	0.544^f^	0.233–0.998	0.049
SBP						
Normal	1			1		
Abnormal	0.626	0.364–1.078	0.091	0.499^g^	0.268–0.928	0.028
DBP						
Normal	1			1		
Abnormal	0.674	0.335–1.355	0.269	0.767^g^	0.351–1.670	0.507
Diabetes						
No	1			1		
Yes	0.278	0.106–0.726	0.009	0.217^g^	0.740–0.639	0.006
Cardiovascular diseases						
No	1			1		
Yes	0.672	0.344–1.312	0.246	0.578^g^	0.255–1.300	0.189
Stroke						
No	1			1		
Yes	3.860	0.504–22.500	0.193	5.529^g^	0.638–39.839	0.121
Myocardial infraction						
No	1			1		
Yes	0.617	0.847–4.490	0.634	0.609^g^	0.562–6.610	0.684
Received protein						
Sufficient intake	**1**			**1**		
Insufficient intake	**0.525**	**0.124–1.230**	**0.140**	**0.238** ^**g**^	**0.027–0.687**	**0.008**
Received fatty						
Sufficient intake	1			1		
Insufficient intake	1.400	0.764–2.570	0.273	1.430^g^	0.679–3.030	0.344
Received carbohydrate						
Sufficient intake	1			1		
Insufficient intake	1.100	0.648–1.880	0.715	0.961^g^	0.518–1.780	0.902
Received fiber						
Sufficient intake	1			1		
Insufficient intake	1.030	0.530–2.00	0.927	0.945^g^	0.417–2.140	0.893
Received magnesium						
Sufficient intake	1			1		
Insufficient intake	1.220	0.725–2.060	0.448	1.170^g^	0.668–2.070	0.572
Received selenium						
Sufficient intake	**1**			**1**		
Insufficient intake	**1.610**	**1.054–2.740**	**0.009**	**2.130** ^**g**^	**1.232–3.098**	**0.018**
Received vitamin A (mcg)						
Sufficient intake	**1**			**1**		
Insufficient intake	**1.150**	**1.001–1.908**	**0.044**	**1.200** ^**g**^	**1.000–2.090**	**0.043**
Received Vitamin E						
Sufficient intake	1			1		
Insufficient intake	0.468	0.110–1.990	0.304	0.672^g^	0.136–3.311	0.625
Received vitamin D (mcg)						
Sufficient intake	1					
Insufficient intake	NR	‐	‐	‐	‐	‐
Received vitamin C						
Sufficient intake	1			1		
Insufficient intake	1.460	0.838–2.570	0.179	1.610^g^	0.814–3.210	0.169

Abbreviations: BMI, body mass index; DBP, diastolic blood pressure; DehPCS, Dehgolan Prospective Cohort Study; SBP, systolic blood pressure.

^a^ Adjusted based on education level, marital status, age, BMI, and waist circumference.

^b^ Adjusted based on sex, marital status, age, BMI, and waist circumference.

^c^ Adjusted based on education level, sex, age, BMI, and waist circumference.

^d^ Adjusted based on education level, marital status, sex, BMI, and waist circumference.

^e^ Adjusted based on education level, marital status, age, sex, and waist circumference.

^f^ Adjusted based on education level, marital status, age, BMI, and sex.

^g^ Adjusted based on education level, marital status, age, BMI, sex, and waist circumference.

## 4 DISCUSSION

The main purpose of this study was to determine the association between nutrients, the infection with COVID‐19, and hospitalization due to this infection. This pandemic has sparked a growth in hypotheses that food products and nutritional supplements are able to “boost” immunity. The immune system is highly complex, consisting of many different cell types and processes, and nutritional adequacy is undoubtedly required to support its function. Specific roles have been established for several micronutrients including vitamins A, C, E, and D, selenium, and zinc, with documented impacts on particular aspects of immune function as a result of clinical deficiencies. Increased susceptibility to infections and poorer outcomes have been commonly observed in cases of undernutrition.^20^, ^21^ The results showed that insufficient intake of selenium as well as vitamins A and E significantly increased the risk of COVID‐19. Also, insufficient intake of vitamin A and selenium significantly increased the hospitalization due to COVID‐19. According to the results of past studies, violation of cellular immune system, phagocytosis function, complement system, cytokine production, and immunoglobulin A antibody secretion were generally related to protein malnutrition and lack of macronutrients or micronutrients. For the proper functioning of the immune system, this system needs a sufficient amount of nutrients (such as fats, proteins, carbohydrates, and micronutrients such as vitamins and minerals). It has been well stated that the absence or deficiency of nutrients due to low consumption or their reduced absorption requires reforms to maintain the proper functioning of the body's immune system.^20^, ^22^, ^23^ Selenium is a powerful antioxidant that fights oxidative stress and protects the body against chronic diseases. High levels of selenium in the blood may even protect the body against some types of cancer. By controlling oxidative stress, selenium reduces the risk of infectious and non‐infectious diseases and is also effective in improving memory.^24^ The results of some studies in the past showed that selenium may also be useful for people with asthma because it reduced inflammations in the body. According to these, it can be claimed that one of the most important benefit of selenium is strengthening the body's immune system.^7^ According to the results of various studies in China, selenium is necessary for the response of the person's immune system against the COVID‐19 virus. Also, German researchers found low levels of selenium in the body were directly related to the increase in deaths caused by the coronavirus. Probably, selenium can be effective in preventing many other viral diseases. Based on this, it can be said that if a person is experiencing selenium deficiency, by taking selenium tablets and compensating for this deficiency, he or she may be able to take an effective step towards preventing the coronavirus from infecting him or her. However, care must be taken when taking selenium supplements, because an excessive increase of this mineral in the body may even lead to brain damage and make a person suffer from the complications of excessive selenium intake. Currently, there are many challenges in determining the association between selenium intake and the prevention of COVID‐19 or the reduction in deaths caused by it, but the proven result is that selenium deficiency increases the incidence of COVID‐19.

Vitamin E, like vitamin C, is a powerful antioxidant that helps the body fight infections. Vitamin E is necessary for about 200 biochemical reactions in the body. Therefore, it is very important for the functioning of the body's immune system.^25^ One of the main properties of vitamin E is that, due to its antioxidant properties, it can protect cells from damage caused by harmful chemicals (called free radicals) usually caused by intracellular metabolism, air pollution, smoking, and ultraviolet rays. Other benefits of vitamin E include its role in strengthening the immune system against bacteria and viruses, especially the coronavirus.^26^

Vitamin A is one of the fat‐soluble vitamins, whose active forms are retinol and retinoic acid. Precursors of these molecules are synthesized by plants in the form of carotenoids.^27^ Vitamin A in the body has various roles, including participation in the biosynthesis of proteins, regulation of oxidative phosphorylation reactions in the mitochondria of liver cells, balance of the mitochondrial membrane, effect on mucous secretions such as tears and spinal fluid, helping in the formation of bone cells, and a fundamental role in sight. This vitamin plays a significant role in improving the body's immune system. The results of various studies showed that this vitamin could affect the reduction in the activity of various infectious viruses, especially the coronavirus, and increase the body's immunity.^28^ Also, published evidences around the worlds, showed that various diets and different micronutrients play a role in the immune system and COVID‐19 patients.^29^, ^30^, ^31^, ^32^

This research was the first study in Iran with this comprehensiveness and a high sample size in order to determine the association between important macronutrients (such as proteins, fats, carbohydrates, and fiber), micronutrients (such as vitamins and antioxidants), the infection of COVID‐19, and hospitalization due to it, which, of course, had several limitations. Among these limitations, we can point out the presence of various confounding factors related to different food and diets, which could not be controlled in the present study. On the other hand, the method of measuring nutrients was self‐reporting, and biochemical tests are suggested to be emphasized in future studies to measure these nutrients.

## 5 CONCLUSION

Based on the results of this cohort study, insufficient intake of selenium and vitamins A and E can significantly increase the incidence of COVID‐19 and hospitalization due to it. Therefore, a proper nutritional protocol is suggested to be developed and distributed in order to receive proper micronutrients and macronutrients during the COVID‐19 pandemic and other infectious disease pandemics. By implementing this protocol, preventing the weakening of the body's immune system against viruses is possible by properly receiving nutrients.

## AUTHOR CONTRIBUTIONS

*Concept development* (provided idea for the research): Yousef Moradi. *Data cleaning*: Dana Vaisi and Yousef Moradi. *Data analysis*: Shadieh Mohammadi, Daem Roshani, and Dana Vaisi. *Supervision*: Yousef Moradi. *Analysis/interpretation*: Farhad Moradpour, Daem Roshani, and Dana Vaisi. *Writing* (responsible for writing a substantive part of the manuscript): Dana Vaisi, Farhad Moradpour, Shadieh Mohammadi, Daem Roshani, and Yousef Moradi.

## CONFLICT OF INTEREST STATEMENT

The authors declare that they have no competing interests.

## ETHICS STATEMENT

The study was a master's thesis in Epidemiology and was financially supported by Kurdistan University of Medical Sciences in Sanandaj, Iran (IR.MUK.REC.1401.125).

## Data Availability

The datasets used and analyzed during the current study are available from the corresponding author on reasonable request.
